# Augmentation of tibial plateau fractures with Trabecular Metal™: a biomechanical study

**DOI:** 10.1186/1749-799X-4-37

**Published:** 2009-09-22

**Authors:** Benoit Benoit, Zhim Fouad, George-Henri Laflamme, Dominique Rouleau, G Yves Laflamme

**Affiliations:** 1 Orthopedic Surgery, Department of Surgery, Université de Montréal Hôpital du Sacré-Cœur de Montréal, 5400, boul. Gouin Ouest, Room C-2080 Montréal, Québec H4J 1C5, Canada; 2 Institut de Génie Biomédical, École Polytechnique, CP6079 Succ. Centre-Ville Montréal, Québec, H3C 3A7, Canada

## Abstract

**Background:**

Restoration and maintenance of the plateau surface are the key points in the treatment of tibial plateau fractures. Any deformity of the articular surface jeopardizes the future of the knee by causing osteoarthritis and axis deviation. The purpose of this study is to evaluate the effect of Trabecular Metal (porous tantalum metal) on stability and strength of fracture repair in the central depression tibial plateau fracture.

**Method:**

Six matched pairs of fresh frozen human cadaveric tibias were fractured and randomly assigned to be treated with either the standard of treatment (impacted cancellous bone graft stabilized by two 4.5 mm screws under the comminuted articular surface) or the experimental method (the same screws supporting a 2 cm diameter Trabecular Metal (TM) disc placed under the comminuted articular surface). Each tibia was tested on a MTS machine simulating immediate postoperative load transmission with 500 Newton for 10,000 cycles and then loaded to failure to determine the ultimate strength of the construct.

**Results:**

The trabecular metal construct showed 40% less caudad displacement of the articular surface (1, 32 ± 0.1 mm vs. 0, 80 ± 0.1 mm) in cyclic loading (p < 0.05). Its mechanical failure occurred at a mean of 3275 N compared to 2650 N for the standard of care construct (p < 0, 05).

**Conclusion:**

The current study shows the biomechanical superiority of the trabecular metal construct compared to the current standard of treatment with regards to both its resistance to caudad displacement of the articular surface in cyclic loading and its strength at load to failure.

## Background

Central depression fractures of the lateral tibial plateau typically occur in elderly osteopenic patients after low-energy injuries [1] accounting for more than 8% of fractures in the elderly. The goal of treating these fractures is to restore the congruity of the articular surface by elevating the depressed articular fragment with impacted bone graft and subchondral lag screw fixation [2,3]. Cancellous bone graft to fill the metaphyseal defect is considered the "Gold Standard"[4]. However, its compressive strength is poor (1-2 MegaPascals (MPa)) which is insufficient to provide significant support to the articular fragments [4,5].

Since loss of reduction remains problematic, many bone substitutes have been suggested to replace the classic autogenous cancellous bone grafting [1,6,7]. Because of their brittleness, most of them are far from human subchondral bone in terms of biomechanical properties [8]. In this study, we compared two similar surgical constructs, the difference between them being the grafting material (autologous cancellous bone vs. Trabecular Metal (TM) Implex/Zimmer, Warsaw, USA).

The purpose of our study is to evaluate the stability and the strength of trabecular metal in the repair of central depression tibial fracture cadaveric model compared to cancellous bone graft.

## Materials and methods

Six matched pairs of fresh frozen human cadaveric tibias were thawed and dissected free. They were osteotomized twenty centimeters (cm) distal to the tibial plateau and potted with a metallic alloy fixture. Each tibia of a pair was randomly assigned to be fixed with the standard construct (two subchondral 4.5 millimeter (mm) cortical screws augmented with cancellous bone graft) or the TM construct (two subchondral 4.5 mm cortical screws augmented with TM). For both constructs; a 2 × 2 × 2 cm defect was created under the lateral tibial plateau, the cartilage with the subchondral bone was divided in four identical pieces to mimic comminution and put back to place after reduction, augmentation and fixation.

**Figure 1:**
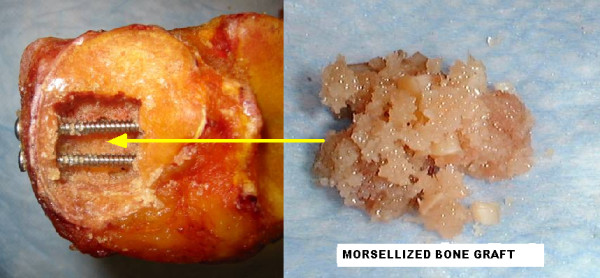
**Standard Construct with impacted cancellous bone graft**.

For the standard construct (Figure 1), cancellous bone graft was impacted in the defect and the two 4.5 mm screws were inserted under the articular surface in order to stabilize the fracture.

**Figure 2:**
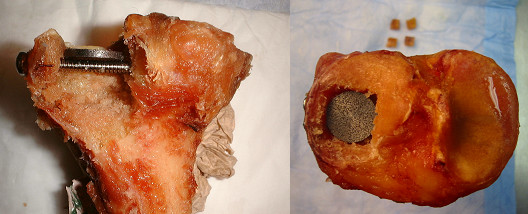
**Trabecular Metal Construct with no graft**.

**Figure 3:**
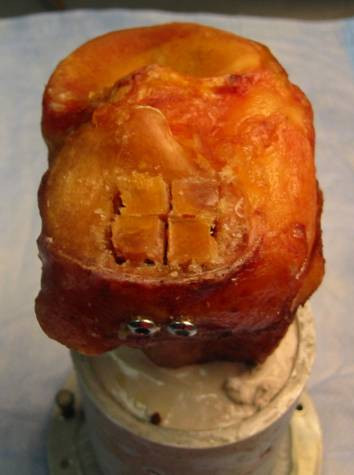
**End result for both construct with anatomic reduction of the osteochondral fragments**.

For the TM construct (Figure 2), tantalum metal with 80% porosity was shaped as a disc (20 mm of diameter by 2 mm of thickness). The TM disc was placed over the two subchondral 4.5 mm screws and under the articular fragments as a subchondral augment with no graft. For both groups, anatomic reduction of the articular surface (Figure 3) was obtained in each specimen.

**Figure 4:**
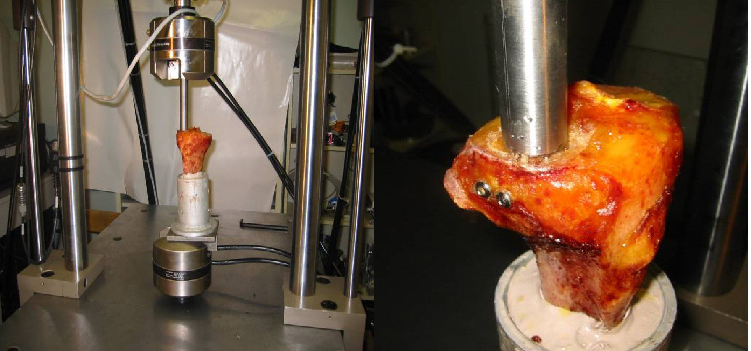
**Diagram of the compression testing apparatus, MTS Bionix™ Test system 858**.

The system used to impose the dynamic and static compression load on the construct is the Bionix™ Test System 810 model (Figure 4), with an MTS Teststar controller.

The depression indentor used to put in charge the fracture was a stainless cylinder (diameter 2 cm). It matched exactly the defect and was applied right on it [5].

### Cyclic loading

The construct was brought up vertically into the MTS system. Each tibia was loaded from 0 to 500 ± 10 Newton (N) five times at a frequency of one hertz for preconditioning. Immediately after preconditioning, 10,000 cycles of compressive load were applied as a sinusoidal waveform from 0 to a peak of 500 ± 10 N at one hertz. During cyclic loading, data was recorded every 10 millisecond. The depression displacement after cycling testing was defined by the linear displacement sensors attached to the MTS machine and its computer.

### Static loading (load to failure)

After cyclic loading, a compressive static load was applied directly to the construct at a rate of 2 mm per minute up to an end displacement of 15 mm. The data acquisition frequency was 50 Hz.

After the load-to-failure test was completed, a description of the fracture was recorded and photographed.

### Statistical analysis

For both mechanical tests, the continuous variables underwent a test for normality and equal variance before parametric statistical analyses. According to cyclic loading test, data were assumed to be normal and equality was achieved in variance. A parametric test (Student *t*-test) was carried out and a two-way analysis of variance was applied. For load to failure test, the normality of the data was not achieved, and a nonparametric test (Mann-Whitney *U *test) was carried out. A minimum significance level of *P *= 0.05 was set for all statistical tests. Statistics were calculated using Excel for Windows.

## Results

**In cyclic loading**, the caudal displacement of the articular surface in each construct varied significantly over the cycles. At 1000 cycles, the displacement was 0.39 ± 0.1 mm in standard construct and 0.33 ± 0.1 mm in the TM construct. No significant differences were detected in either the standard or the TM construct for the first 1000 cycles. Following this period, the displacement was significantly increased in both constructs. Maximum mean displacement was 1.32 ± 0.1 mm [1.01 to 1.51 mm] at 10,000 cycles for the standard construct group and 0.80 ± 0.1 mm [0.73 to 0.95 mm] for the TM construct group (Student *t*-test, *P *< 0.05).

**Figure 5:**
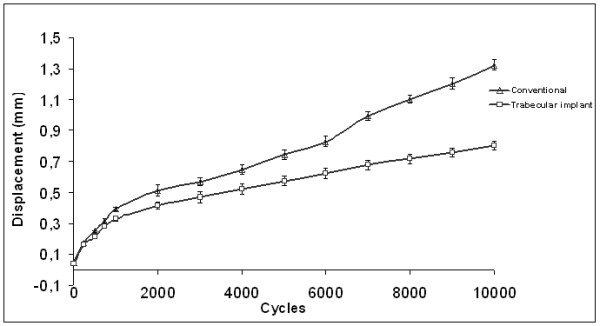
**Dynamic Loading**: Average depression in the constructs during cyclic loading. Maximum mean displacement was 1.32 ± 0.1 mm [1.01 to 1.51 mm] for the standard construct group and 0.80 ± 0.1 mm [0.73 to 0.95 mm] for the TM construct group (*P *< 0.05).

Figure 5 shows the curves for mean caudal displacement of the articular surface in both constructs. The two curves were monotone and presenting a positive slope. Cyclic stability behaviour is not thoroughly influenced by cyclic loading.

**Figure 6:**
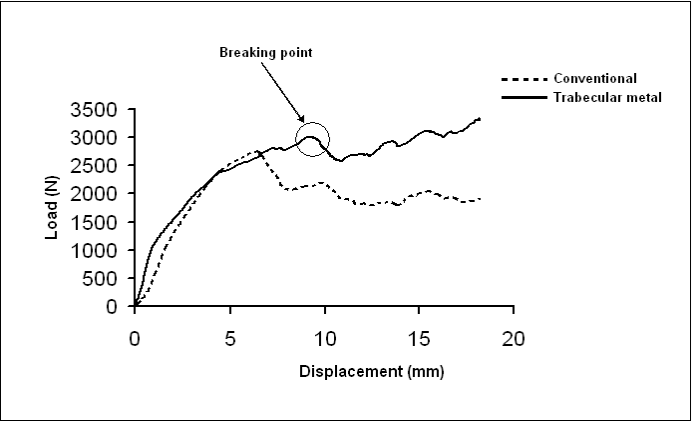
**Static Loading**: Load versus displacement curves for specimens with trabecular implant and conventional technique.

**In static loading**, the failure properties of each constructs were found to be sensitive to the type of mechanical testing (Figure 6). Interestingly, the mean load at failure for the TM construct was significantly higher than that of the standard construct (3275 N vs. 2650 N) (Mann-Whitney *U *test, *P *< 0.05). Moreover, the stability in the TM group was also greater (362 *vs*174 Newton per millimeter (N/mm)) (Mann-Whitney *U *test, *P *= 0.02). No significant difference was noted in the mechanism of failure for both types of constructs. In every construct, no hardware failure was noted. In gross appearance, the lateral tibial plateau lost height progressively under loading until a fracture occurred around the screws.

No hardware failure was observed in either construction. The TM disc maintained its pre-testing appearance in all cases.

## Discussion

Depression type lateral tibial plateau fractures are usually low-velocity injuries that result from axial and bending forces across the knee. Articular surface depression is more frequently seen in elderly patients because of a progressive weakness in the subchondral cancellous bone secondary to osteoporosis [6]. The goal of treating these fractures is to restore the congruity of the articular surface. This is commonly done by elevating and realigning the depressed articular surfaces, placing a graft material into the metaphyseal defect and supporting this reconstruction with internal fixation [1]. The "gold standard" in bone grafting is autograft cancellous bone. The major problems with autologous bone grafts are donor site morbidity and poor compressive strength. Its inability to provide significant support to the articular fragments has lead to a high incidence of loss of correction and malunion [1].

In an attempt to improve stability, many recent studies have focused on specific implants to allow better fixation. Benirschke and Swiontkowski [1] in 1993 reported on the use of 3.5-mm small fragment t-plates to decrease the bulk of the hardware. Twaddle et al [3] in 1997 compared a low-profile subchondral raft construct (small fragment fixation) with conventional large fragment fixation in a lateral plateau fracture model. In this study, the raft plate fixation allowed significantly less displacement under axial loading than the buttress plate construct (2954 versus 968 N/mm). They suggested that a raft of screws might provide superior support of the articular surface. Screw pullout strength in the proximal tibia has been studied by Westmoreland et al [9] in 2002. They found no difference between 6.5-mm, 4.5-mm and 3.5-mm screws tested in the metadiaphyseal proximal tibia. Their study supports the use of small-fragment fixation in the treatment of tibial plateau fractures. Recently, Karunakar et al [2] showed that a subchondral raft of screws may provide superior resistance than traditional fixation constructs for local depression loads. Smaller screws placed closer to the subchondral bone provided greater resistance to local depression loads without compromising overall construct stiffness. Most importantly, they showed that the addition of cancellous bone graft did not significantly increase the stiffness of a conventional large fragment construct.

A multitude of bone substitutes have been suggested to support the depressed articular fragments of tibial plateau fractures [5,10-15]. The most widely used in the trauma setting is a calcium phosphate cement that has been shown to be biocompatible and osteoconductive. Recent clinical studies have compared calcium-phosphate cement to conventional autogenous iliac bone graft (AIBG) considered the gold standard [4]. The calcium phosphate was superior in all these trials since AIBG has very weak compressive strength (1-2 MPa). In an animal study [11], a calcium phoshate cement (Norian SRS, Norian Corporation, Cupertino, CA, USA) was considered by the authors to be an attractive competent augmentation material for repair of compromised metaphyseal bone. No significant difference was found between SRS Norian and morsellized bone allograft; although the trend suggested that the tibias treated with allograft had faster incorporation with faster return to normal strength.

Calcium phosphate cements may have the compressive strength similar to cancellous bone, but their brittleness offers poor resistance to fragmentation and poor fatigue resistance [8]. In another study evaluating calcium phosphate bone cement in a central depressed tibial plateau fracture cadaveric model [5], the average depression of the articular fragment was not significantly different than the standard treatment with bone graft with screws. To obtain significant results, extensive curetting of the cancellous bone under the subchondral bone plate was needed.

The ideal substitute for the metaphyseal defect is still unknown. TM is a porous tantalum metal structure that has the appearance of cancellous bone and similar biomechanic properties [16]. Tantalum can be considered the most biocompatible biomaterial and the most resistant to the corrosion phenomena. It is characterised by its strength, low stiffness, and resistance to fatigue failure. The internal microstructure of porous tantalum metal consists of interconnected pore network that allows a biological attachment to the bone and the regeneration of the new bone. Due to its porosity, TM has an elastic modulus similar to that of subchondral bone. Its open cell structure has interconnecting pores resulting in a construct highly porous (80%) that is resistant to fatigue failure and that will maintain its strength for the duration of the healing process [16]. The mechanical properties do not degrade with time or cyclic loading as seen with calcium phosphate cement.

The concept behind the design of a TM disc evolved from the mode of failure seen when testing tibial plateau fracture in vitro. Load-to-failure experiments showed the cancellous bone adjacent to the subchondral plate to be the weakest component of the fracture constructs [5]. This phenomenon demonstrates that the fracture construct is only stable as the foundation on which it rests. Restoring the subchondral plate is the key structure allowing optimal stability and improved load transfer.

The current study shows that TM augmentation of a raft screw construct is biomechanically superior to cancellous bone graft. The articular displacement when submitted to 10000 cycles was reduced by 40% and its stiffness at load to failure was significantly improved. The strengths of this study were that a worst case scenario was recreated by applying direct loading with an indentor to the fracture site and only the graft material differed between the two groups studied (AIBG vs TM). However, the TM trabecular metal is a disc-shaped solid construct thus it was expected to have better loading resistance when compared to the AIBG standard group with morsellized bone graft. The shape of the specimen tested differs completely since the tantalum used for this test was a TM disc implant rather than morsellized pieces like the bone graft. In the clinical setting, the lateral femoral condyle would distribute the load more evenly over the plateau to protect the reduction^5^. The soft tissues and the meniscus would also bear a significant part of the load applied. This study has some limitations: the fracture created was reproducible among our specimens but possibly different from an in-vivo pure depression lateral tibial plateau fracture and the absence of soft tissues also facilitated the optimal placement of the implants. Furthermore, bony ingrowth is a critical factor that plays an important role on influencing the mechanical properties of TM. Progressive fracture healing is neglected in this biomechanical model. Animal studies or clinical studies are needed to further evaluate TM as an alternative to graft.

## Conclusion

Despite the aforementioned limitations, we think that the results of this study suggests that the use of a subchondral augmentation with TM in the treatment of lateral tibial plateau fractures could better resist the compressive forces across the fracture site in the early postoperative period preventing loss of reduction. Porous tantalum with mechanical properties and pore network similar to cancellous bone can have potential applications in orthopaedic trauma surgery. Further study will be needed to evaluate the feasibility of surgical application in the clinical setting.

## Competing interests

The authors declare that they have no competing interests.

## Authors' contributions

1) BB, GYL, have made substantial contributions to conception and design, or acquisition of data, or analysis and interpretation of data;

2) BB, DR, GHL, GYL have been involved in drafting the manuscript or revising it critically for important intellectual content; and

3) BB, GHL, DR, GYL have given final approval of the version to be published.
